# Factors affecting health insurance utilization among insured population: evidence from health insurance program of Bhaktapur district of Nepal

**DOI:** 10.1186/s12913-023-09145-9

**Published:** 2023-02-15

**Authors:** Sushmita Ghimire, Sailaja Ghimire, Pratik Khanal, Reshu Agrawal Sagtani, Sudarshan Paudel

**Affiliations:** 1 School of Public Health, Patan Academy of Health Sciences, Lagankhel, Lalitpur, Nepal; 2 Nepal Public Health Association, Lalitpur, Nepal

**Keywords:** Health insurance, Insured, Nepal, Service utilization

## Abstract

**Background:**

The Government of Nepal introduced the family-based health insurance program in 2016 to increase financial protection and improve access to health care services. The study aimed to assess factors associated with the utilization of health insurance among the insured population in an urban district of Nepal.

**Methods:**

A cross-sectional survey using face-to-face interviews was conducted in 224 households in the Bhaktapur district of Nepal. Household heads were interviewed using a structured questionnaire. Logistic regression with weighted analysis was done to identify predictors of service utilization among the insured residents.

**Results:**

The prevalence of health insurance service utilization at the household level in the Bhaktapur district was 77.2% (n = 173/224). The number of elder members in the family (AOR 2.7, 95% CI 1.09–7.07), having a family member with chronic illness (AOR 5.10, 95% CI 1.48–17.56), willingness to continue health insurance (AOR 2.18, 95% CI 1.47–3.25) and membership duration (AOR 1.14, 95% CI 1.05–1.24) were significantly associated with the utilization of the health insurance at the household level.

**Conclusion:**

The study identified a particular group of the population who were more likely to utilize health insurance services, including the chronically ill and elderly. Health insurance program in Nepal would benefit from strategies to increase population coverage in health insurance, improve the quality of health services, and retain members in the program.

## Background

The movement towards achieving Universal Health Coverage (UHC) is mounting attention worldwide, and health insurance has been instrumental in this attempt. Health insurance with a prepaid mechanism ensures the risk pooling and redistribution of financial resources to secure financial protection against treatment costs [1]. Many countries in the world have established the principle of UHC via social health insurance (SHI) [2]. The SHI has a substantial potential for improving financial protection by reducing out-of-pocket payments and enhancing health care utilization among the insured population by promoting social inclusion in health care.

The insured citizens in countries with strong health care systems have improved health outcomes due to access to prompt health care and consequently suffer the less financial burden [3]. Many countries like Germany, United Kingdom, South Korea, and Thailand were able to obtain full coverage of their population through an effective health insurance system [4]. However, low and middle-income countries (LMICs) have rarely utilized this approach and mostly depend on general revenues and direct out-of-pocket payments (OOP) for seeking health care [5]. Even in countries where health insurance has been implemented, the scheme is performing poorly than anticipated leading to the wastage of resources and loss of trust among the enrolled members [3].

The Government of Nepal has the vision to improve the health of all Nepalese people by increasing their access to healthcare services with a national health insurance program (NHIP) as an important strategy [6]. Nepal’s National Health Policy 2019 has envisioned providing specialized services to the population through health insurance while ensuring basic health services through general taxation [7]. The implementation of NHIP in Nepal was started in April 2016 by enrolling the informal sector (household level) at the district level [8, 9]. According to the annual report for the fiscal year 2020/21, 4.5 million people have been enrolled in NHIP in Nepal, and the geographical coverage is 75 out of 77 districts [10]. The Health Insurance Board (HIB) - the government purchasing agency has set a flat contribution amount of Nepalese Rupees (NPR) 3,500 (around 30 USD) for a five-member household with an additional contribution amount of NPR 700 (about 6 USD) for each additional member. The government has provided a full waiver in contribution amount for elderly above 70 years and family members of ultra-poor households (poor households identified in 26 out of 77 districts), people living with HIV, drug-resistant tuberculosis patients, leprosy patients, and those with complete disability [11]. The benefit package is comprehensive and covers outpatient, inpatient, and emergency services and 1,133 types of drugs. However, the benefits package is limited to a ceiling of NPR 100,000 (around 900 USD) for a five-member family. An additional benefit of NPR 20,000 (about 180 USD) is covered for each additional member. A total of 440 service sites registered with the HIB provide service, including primary health centers, government hospitals, and private and community hospitals [11, 12].

In the early stage of implementing health insurance, factors associated with enrolment, drop out and service utilization are important. Assessment of these factors assists in timely improvement in the performance of the NHIP as well as revising current implementation modalities. Existing studies and programmatic reviews have suggested that Nepal’s NHIP is marked by low enrolment, high dropout, poor trust in health services, limited availability of drugs at health facilities, and high provider payment concerning the contribution amount collected [13–15]. However, a research gap exists in understanding the factors affecting service utilization among insured members in Nepal. Most of the research in Nepal solely looked at the variables influencing health insurance program enrollment. Only a few studies have been carried out globally and in Nepal that has identified factors affecting the service utilization of insured people [16, 17]. In this context, this study aims to identify the factors associated with the utilization of health insurance services among the insured population.

## Methods

### Study design and settings

A cross-sectional household-based study was conducted in the Bhaktapur district of Nepal. Bhaktapur is an urban area and is one of the three districts of Kathmandu valley; the other two are Lalitpur and Kathmandu. Bhaktapur district consists of four municipalities which further comprise a total of 38 wards-the lowest administrative unit. Enrolment in health insurance started in the district in June 2017. The households enrolled till December 2018 were included in the sampling frame. Out of a total of 68,557 households, 16,623 were enrolled in health insurance. The district of Bhaktapur was purposefully chosen as one of the districts to enroll in health insurance during the second phase of expansion.

### Operational definition

#### Insured household

The family who ever enrolled in the Health Insurance Program till December 2018 (6 months before data collection).

#### Insurance service utilization

Those families who have ever utilized or claimed health insurance services, after getting a valid card.

#### Membership duration

The time duration since getting a membership card in the program.

### Sample size and sampling method

We calculated the sample size using the formula for the cross-sectional survey [18]. The proportion of health insurance service utilization was taken as 9.7% considering the proportion of the population enrolled in health insurance in the country in 2016/17. According to the annual report, 22,228 out of 228,113 insured population have utilized health services through health insurance [19].

The following parameters were considered while calculating the sample size;

Prevalence (p) = 9.7%.

Complement of P (q) = 90.3%.

Margin of error (α) = 5%.

Non response rate = 10%.

Design Effect (DE) = 1.5.

Minimum Sample Size (N) = z^2^pq/e^2^ *(1 + Non response rate).

= {(1.96)^2^ 0.097*0.903}/(0.05)^2^ *(1 + 0.1)*design effect.

= 222.

The required minimum sample size for the study was 222. We adopted two-stage probability sampling for the selection of households for the study. Firstly, we randomly selected four out of 38 wards by using the population proportionate to size (PPS) sampling technique for which the list of households enrolled in health insurance at the ward level was obtained from the HIB district office. The cumulative sum of the households enrolled was then determined after creating separate columns for the wards and enrolled households. The number of wards to be sampled was decided to be 10% of the total 38 wards (i.e., 4 wards). The sampling interval was then calculated by dividing the total number of households in the 38 wards by the number of wards to be a sample (i.e., 4). The random start was identified by selecting a random number between 1 and the sampling interval. As a result, the wards were chosen based on the sample interval.

The total sample size was then distributed equally to the selected wards. Since the minimum sample size was 222, a total of 56 households were taken from each ward resulting in a total sample of 224 households. Thereafter, a systematic random sampling technique was done to select households from the selected wards. The sampling frame of the households in the selected wards ranged from 394 to 822 households. The sampling interval was identified and the household was selected after a fixed periodic interval with a random starting point. As health insurance is a family-based program, household head of the household was selected for the interview.

### Study variables

The dependent variable of the study is health insurance service utilization while the independent variables are background and mediating variables. The study variables of this study are presented in Table 1.

**Table 1:** Study variables

S.N.	Variables	Categories of variables
**A**	**Dependent variable**	
1	Utilization of health insurance	Yes, No
**B**	**Background variables**	
1	Age of household head	Years
2	Gender of household head	Male, Female, Others
3	Ethnicity	Dalit, Janajati, Madhesi, Muslim, Brahmin/ Chhetri, Others (based on Health Management Information System classification)
4	Literacy	Illiterate, Literate
5	Family size	Number
6	Family type	Nuclear, Joint Family /Extended
7	Number of children in a family	Number
8	Number of elderly (≥ 60 years of age) in the family	Number
9	Average annual income	Amount
10	Major source of expenditure	Household general expenditure, Health, Education
11	Expenditure in Health	Amount
12	Socio economic status	Upper, middle, lower (calculated by using Modified Kuppuswamy scale [42]
**C**	**Mediating Variables**	
1	Conditions of seeking health care	Regular during illness, Only after not responding to other treatment, Only in emergency / severe condition, Regular check-up, Other
2	Presence of chronic illness	Yes, No
3	Presence of family member with a disability	Yes, No
4	Comprehensive knowledge in health insurance	Yes, No
5	Current membership status	Currently insured (has valid ID card), Previously insured (non-renewal)
6	Enrolled in other modes of health insurance	Privately purchased commercial insurance, Health Insurance through the employer
7	Last annual contribution amount paid	Amount
8	Total family members enrolled in health insurance	Number
9	Willingness to continue	Yes, No
10	Membership Duration	Months

### Data collection

The field survey was carried out from September 2019 to November 2019. The researchers visited the sampled households which were identified with the help of the district HIB office and enrolment assistants. A face-to-face interview was done with the household head of the family after obtaining written informed consent. Data collection was done by using the semi-structured questionnaire divided into three parts: socio-demographic characteristics, knowledge about health insurance and membership, and insurance service utilization. The data collection tool was developed based on a rigorous literature review. The face and content validity were maintained by consulting with the experts. The precision of the data was ensured by closely examining the relevancy of the questionnaire to the objectives of the study. The questionnaire was pre-tested before administration for local validity and reliability. The questionnaire’s accuracy in terms of content structure, ambiguous terminology, unclear questions, and language has been analyzed and updated in response to the expert’s comments and suggestions. The questionnaire was translated into the Nepali language for collecting the data which was further back-translated to ensure the validity of the tool. Investigators frequently kept a careful observation of the data collection procedure to make sure that it was complete, accurate, and consistent.

### Data analysis

Data were entered, coded, and edited in Epi Info 7. The cleaned data were then analyzed using the STATA 13 software. Firstly, we did a bivariate analysis between the dependent and background variables. Initially, in bivariate analysis, a single variable at a time was entered; unadjusted OR and 95% CI were computed for all independent variables. The p-value from the bivariate analysis was checked and the p-value less than 0.25 were taken to the multivariable model. The variables that showed significant association in bivariate analysis: ethnicity, literacy, type of family, number of elder members in the family, and expenditure in health, were included in a multivariable logistic regression model. Likewise, we conducted a bivariate analysis between the dependent variable and the mediating variables. The variables that showed significant association at a 25% level of significance: family members with chronic illness, family members with comprehensive knowledge of health insurance, last annual premium paid, willingness to continue, and membership duration, were also included in multivariable logistic regression analysis.

Further, we checked the multicollinearity test through variance inflation factor (VIF) among all the variables which were eligible for multivariate logistic regression [20, 21]. Those variables showing VIF less than two were only taken for regression analysis [22]. All the variables except ethnicity showed VIF less than two and were fitted in the final multivariable logistic regression model. We set the level of significance at 5%.

## Results

### Background characteristics of participants

Among the total 224 participants, 83% of the household heads were male. The median age of the household head was 54 years. Similarly, 83% of household heads were literate, two out of three had nuclear families, and the average family size was 5.6. Among the total households, 45% of households had elderly members in the family, and a quarter of households had children below five years in their family. Nearly half of the families’ major expenditure was on household general expenses, while 30.8% and 24.1% of the participants reported that their major expenditure was on education and health, respectively. On average, one-fifth (20.4%) of their income was spent on health. While assessing the family’s socioeconomic status, more than half of the participants had lower socioeconomic status. The proportion of households having at least one member with chronic illness in their family was 56.3% and among those, nearly two-thirds (64.3%) had a member with chronic illness in the family. Around 3% had a member with a disability in their family.

The socio-demographic characteristics of the study participants are presented in Table 2.

**Table 2:** Socio-demographic characteristics of the study participants (N = 224)

Variables	Frequency		Percentage	
**Mean age (± SD) of household head** 54 0.8 (± 13.12) years				
**Gender of Household head**				
Male	192		85.7	
Female	32		14.3	
**Ethnicity**				
Janajati	150		67.0	
Brahmin/ Chhetri	65		29.0	
Dalit	9		4.0	
**Literacy Status**				
Literate	188		83.9	
Illiterate	36		16.1	
**Types of Family**				
Nuclear Family	152		67.9	
Joint and Extended	72		32.1	
**Average member (± SD) in family** 5.6 (± 1.93)				
**Elderly member in the family**				
No	123		54.9	
Yes	101		45.1	
**Number of elderly members in the family (n = 101)**				
1	58		57.4	
2	39		38.6	
3	3		3.0	
4	1		1.0	
**Children in Family**				
No	167		74.6	
Yes	57		25.4	
**Number of children in the family (n = 57)**				
1	47		82.5	
2	9		15.8	
3	1		1.8	
**Chronic illness in the family**				
No	98		43.7	
Yes	126		56.3	
**Number of family members with Chronic Illness in the family (n = 126)**				
1	81		64.3	
2	38		30.2	
3	7		5.6	
**Family members with a disability**				
No	218		97.3	
Yes	6		2.7	
**Major expenditure**				
Household	101		45.1	
Education	69		30.8	
Health	54		24.1	
**Socioeconomic status of household**				
Lower	126		56.3	
Middle	95		42.4	
Upper	3		1.3	
**Average family annual income = NRS** 553,200				
**Average expenditure in health (in percentage) =** 20.4 ± 16.2				

### Knowledge about health insurance

Comprehensive knowledge about health insurance services was referred to as knowing about contribution amount; benefit ceiling, time of renewal, and service availability. Less than one in five (18.8%) of the participants had comprehensive knowledge about health insurance services (Table 3).

**Table 3:** Distribution of insured residents having comprehensive knowledge about Health Insurance in Bhaktapur District

Knowledge on health insurance	Frequency	Percentage
Knowledge of contribution amount		
Yes	45	20.09
No	179	79.91
Knowledge of maximum benefit ceiling		
Yes	169	75.45
No	55	24.55
Knowledge of the time of renewal		
Yes	199	88.84
No	25	11.16
Knowledge of service available		
Yes	220	98.21
No	4	1.79
Comprehensive Knowledge on health insurance		
Yes	42	18.75
No	182	81.25
Total	224	100

### Membership status and utilization of health insurance

Among study participants, 11.2% (n = 25/224) had dropped out of the health insurance program. The majority (91.5%, n = 205/224) of the study participants were willing to continue their membership in the health insurance program. The household proportion of utilization of health insurance services was 77.2% (n = 173/ 224).

### Factors associated with the utilization of health insurance among households

In the adjusted analysis of the association between the dependent and background variables, the number of elder members in the family showed a statistically significant association with service utilization of health insurance benefits. Three mediating variables—the presence of chronic illness in the family, willingness to continue, and membership duration—were also significantly associated with the service utilization of health insurance benefits. 

After including variables that showed significant association in the final regression model, an increase in the number of elder members in the family had higher odds (AOR = 2.70; 95% CI: 1.09–7.07) for service utilization. Likewise, the family having a member with chronic illness was five times more likely (AOR = 5.10; 95% CI: 1.48–17.56) to utilize services compared to the family having no members with chronic illness. Additionally, the utilization of health insurance services increased by 2.1 times among those willing to continue in the health insurance program (AOR 2.18; 95% CI: 1.47–3.25) than those who did not want to continue the membership. Similarly, with the increase in the number of months of membership duration in the health insurance program, the utilization of health insurance services was also significantly higher (AOR 1.14; 95% CI: 1.05–1.24) (Table 4). The variables such as literacy status of household head, types of family, expenditure in health, knowledge about health insurance, and last annual contribution amount paid didn’t show any significant association in the final regression model.

**Table 4:** Multivariate Analysis of factors associated with the utilization of health insurance services

Variables	Category	Utilization of health insurance (n = 173) N (%)	OR (95% CI)	P value	AOR (95% CI)	P value
**Literacy**	Illiterate	30 (17.3%)	Ref	0.225	Ref	0.25
	Literate	143(82.7%)	0.63 (0.24–1.64)		1.75 (0.48–6.3)	
**Types of family**	Joint/Extended	58 (33.5%)	Ref	0.175	Ref	0.141
	Nuclear	115(66.5%)	0.75 (0.44–1.25)		1.64 (0.74–3.63)	
**Number of Elderly**			2.86 (2.39–3.43) *	0.0003	2.7 (1.09–7.07) *	0.040
**Expenditure in Health**			1.02 (0.98–1.06)	0.1106	1.00 (0.96–1.05)	0.658
**Presence of Chronic Illness**	No	57 (32.9%)	Ref	0.002	Ref	0.025
	Yes	116(67.1%)	8.3 (4.32–16.09) *		5.10 (1.48–17.56) *	
**Knowledge about health insurance**	No	136 (78.6%)	Ref	0.182	Ref	0.200
	Yes	37 (21.4%)	2.44 (0.47–12.7)		2.02 (0.51–8.02)	
**Last annual contribution amount Paid**			1.00 (1.00–1.00)	0.0430	1.00 (0.99-1.00)	0.112
**Willingness to continue**	No	12(6.9%)	Ref	0.101	Ref	0.008
	Yes	161(93.1%)	2.13 (0.76–5.99)		2.18 (1.47–3.25) *	
**Membership duration**			1.15 (1.05–1.24)	0.0128	1.14(1.05–1.24) *	0.013

* statistically significant at p < 0.05

### Reasons for non-utilization of health insurance services

The most common reasons for not utilizing health insurance services were having no health problems (22.99%), seeking other treatment (22.99%), and hearing about poor experiences of utilizing insurance services from other service users (19.54%). In addition, participants reported other reasons as long waiting lines in the health facility, bothersome procedures to get treatment, lack of time, and being unaware of where to go for treatment, as the reasons for the non-utilization of health insurance (Table 5).

**Table 5:** Reasons behind non-utilization of Health Insurance among insured residents in Bhaktapur district, (N = 51)*

Reasons for non-utilization	Frequency (n = 87)	Percentage
No Health problem (No need)	20	22.99
Seek other treatment	20	22.99
Heard bad news about service delivery	17	19.54
Do not like the staff at the health facility	8	9.20
Do not address the health need by services	6	6.89
Long distance to the health facility	2	2.30
Other	14	16.09

* Multiple responses

## Discussion

This study investigated the factors associated with health insurance service utilization at the household level in a urban district. Our study showed a higher number of elderly members in the family was significantly associated with the utilization of health insurance services. Similar findings were observed in a study done in China and Taiwan [23, 24]. An increase in age elevates the vulnerability of getting ill health, which leads to generating more health needs, resulting in high utilization of health care benefits [25]. Another reason for high utilization could be due to the full waiver in contribution amount for the elderly above 70 years in NHIP. This could motivate them to utilize health insurance services.

Households with members suffering from chronic illness were also more likely to utilize health insurance services in this study. The higher proportion of service utilization might be because of increased healthcare needs for chronically ill people. A similar finding was observed in a previous study done in three districts of Nepal, which showed higher service utilization among patients suffering from chronic illnesses like diabetes and hypertension after the program’s inception in the district [26]. Also, families with chronically ill people were more likely to join the health insurance program, as evident from a study done in Illam, Nepal [9]. Studies from the Community Based Health Insurance (CBHI) program of India [25] and rural China however [27, 28] did not show any association between the presence of chronic illness and health insurance utilization. In our context, a higher tendency of health insurance service utilization among chronically ill people exists. The government should address the possible selection bias by increasing population enrolment in health insurance and ensuring a larger risk pool for the financial sustainability of the health insurance program [29].

Our study showed a significant association between health insurance utilization and membership duration of the health insurance program. The possible reason might be that members with longer duration are aware of the benefits of the health insurance program. However, the membership duration showed no association with the utilization of health insurance benefits of out-patients services in the Vietnam household living standard survey [30]. Another study in St. Louis, USA revealed a reduction in utilization rates with increased membership duration over five years. The reasons behind this were due to centralizing health care system and long waiting lines which involved travel and time cost [31].

Additionally, willingness to continue the program was a significant predictor for health insurance utilization in this current study. The possible explanation for this might be the insured’s positive experience in getting health insurance benefits. High dropout rates put challenges in the reduction of insurance pool size and the negative impact on the new enrollment rate. Around 90% of the participants were willing to continue the program in the future. Given that the prevalence of service utilization was 77.2%, this means some households are not using the service, but are willing to continue the program which is a positive sign. The figure is consistent with the study conducted in three districts (Kailali, Baglung, and Illam) of Nepal where over 90% of insured groups expressed their willingness to membership renewal and recommend their friend to the health insurance program [26]. Similar findings were identified in a study in Ethiopia in which almost insured households (96%) said they would renew their membership to the insurance program [32]. Literature has demonstrated that the decision to continue in the program reflects the individual’s risk aversion and demand for certainty as the certainty level regarding relatively good health reduces acceptance for insurance uptake and vice versa [33–35].

In our study, the family’s education status, socioeconomic status, and comprehensive knowledge regarding health insurance were not significantly associated with service utilization. This study also examined the prevalence of the utilization of health insurance programs, which was 77%. The dropout rate was measured as 11%, which was very low compared to the national rate, i.e., (44.5%) [13]. Although the overall performance indicator was remarkably good compared to the national rates, the comprehensive knowledge on health insurance (18.8%) was lower than in the study conducted in two districts (Baglung and Kailali) of Nepal where health insurance was first implemented. According to the study, 72% of respondents are aware of health insurance [36].

The study showed various reasons for the non-utilization of the health insurance service which included not being ill, seeking other treatment, and hearing about previous bad experiences from the service users. We identified similar reasons for the non-utilization of health insurance services in studies conducted in different countries [37–41].

The study has some limitations. The cross-sectional study was conducted in an urban setting, thus, the findings derived from this study cannot be generalized to the whole country. Similarly, the study might have encountered respondent bias despite the study team’s effort to explain the purpose of the study. Nevertheless, this study is the first of its kind in Nepal, exploring the factors affecting the utilization of health insurance among the insured population.

## Conclusion

The prevalence of utilization of health insurance services at the household level was 77.2%. The number of elder members in the family, presence of chronic illness in the family, willingness to continue, and membership duration were significantly associated with the utilization of health insurance services. As families with elderly and chronically ill members have more tendency to utilize health insurance services, we recommend the HIB focus on increasing population coverage in health insurance for adequate risk pooling and ensuring the financial sustainability of NHIP. This can be done by expanding health insurance to the formal sector and disadvantaged population, setting contribution amounts based on the equity in financial burden, revising benefit packages based on priority setting, and increasing responsiveness among health institutions. Similarly, policymakers should focus on the strategies to retain the existing member in the health insurance program and implementation of health insurance literacy programs targeting the community people. The findings guide stakeholders to redesign the existing health insurance scheme, which is voluntary and family-based and with limited risk pooling. The findings could be important for similar settings to set up strategies in the perspective of scaling up health insurance coverage and adherence.

## Data Availability

The datasets used and/or analyzed during the current study are available from the corresponding author upon reasonable request.
